# A Photocytes-Associated Fatty Acid-Binding Protein from the Light Organ of Adult Taiwanese Firefly, *Luciola cerata*

**DOI:** 10.1371/journal.pone.0029576

**Published:** 2011-12-29

**Authors:** King-Siang Goh, Chia-Wei Li

**Affiliations:** Institute of Molecular and Cellular Biology, National Tsing-Hua University, Hsinchu, Taiwan; Cardiff University, United Kingdom

## Abstract

**Background:**

Intracellular fatty acid-binding proteins (FABPs) are considered to be an important energy source supplier in lipid metabolism; however, they have never been reported in any bioluminescent tissue before. In this study, we determined the structural and functional characteristics of a novel FABP (lcFABP) from the light organ of adult Taiwanese firefly, *Luciola cerata*, and showed anatomical association of lcFABP with photocytes.

**Principal Findings:**

Our results demonstrated the primary structure of lcFABP deduced from the cDNA clone of light organ shares structural homologies with other insect and human FABPs. *In vitro* binding assay indicated the recombinant lcFABP binds saturated long chain fatty acids (C_14_-C_18_) more strongly than other fatty acids and firefly luciferin. In addition, tissue distribution screening assay using a rabbit antiserum specifically against the N-terminal sequence of lcFABP confirmed the light organ-specific expression of lcFABP. In the light organ, the lcFABP constituted about 15% of total soluble proteins, and was detected in both cytosol and nucleus of photocytes.

**Conclusions:**

The specific localization of abundant lcFABP in the light organ suggests that sustained bioluminescent flashes in the light organ might be a high energy demanding process. In photocytes, lcFABP might play a key role in providing long chain fatty acids to peroxisomes for the luciferase-catalyzed long chain acyl-CoA synthetic reaction.

## Introduction

**Figure 1:**
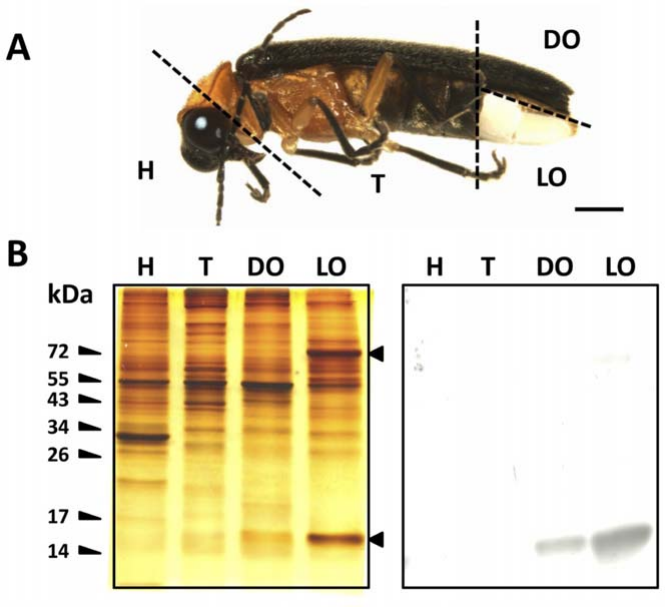
Analysis of the tissue-specific distribution of lcFABP in an adult firefly, *L. cerata*. A) Morphology of a male *L. cerata.* B) Tissue supernantants were prepared from homogenized body segment of head (H), thorax (T), dorsal organ (DO) and light organ (LO). Total of 15 µg proteins of each homogenate was separated using a 15% acrylamide gel, followed by silver nitrate staining (B. left panel) or western blotting using lcFABP-specific rabbit antiserum (B. right panel). Two light organ-specific proteins are indicated by the arrowheads toward left side. Molecular weight markers are indicated as kDa. Scale bar, 1 mm.

Adult fireflies (Coleoptera: Lampyridae) possess a specialized abdominal light organ or lantern (Fig. 1A) to generate continuous flashes of bioluminescence for sexual communications [1], [2]. The light organ is a slab-like tissue composed of a ventral photogenic layer and a dorsal reflector layer. The photogenic layer is assembled by a group of light-producing cells called photocytes, and an extensive nerve-connected tracheal system [3], [4]. The reflector layer consists of cells filling with white granules - presumed to be urate in nature; it is considered to reflect the light emitted from the photogenic layer for increasing light intensity [3], [4].

Despite the extensive studies over a century, the occurrence of bioluminescence in the light organ is still poorly understood [4]. To date, only three light organ proteins have been well characterized for their roles in firefly bioluminescence, including luciferase, luciferin-regenerating enzyme and nitric oxide synthase [5]–[8]. Luciferase is the key enzyme that catalyzes the light emitted from luciferin in the presence of ATP, Mg^2+^ and oxygen [5]. Luciferin-regenerating enzyme, a 38 kDa protein, acts to convert the bioluminescent reaction product, oxyluciferin, back to luciferin [6]. Nitric oxide synthase is essential for controlling the pattern of bioluminescent flashes [7], [8].

How adult fireflies utilize cellular energy during sustained flashes is still a mystery. The light organ of adult fireflies is differentiated from fat body during development [9], and was reported lacking the carbohydrate-metabolic enzyme activities [10]. Crucially, a previous study indicated that firefly luciferases might be involved in the initial step of lipid metabolism [11]. These evidences implied that lipid is the most important source of energy for firefly bioluminescence.

Intracellular fatty acid-binding proteins (FABPs) are a group of small soluble proteins (14∼15 kDa) that can non-covalently bind to saturated or unsaturated long chain fatty acids (≥14 carbons) with high affinity. These proteins belong to a large multigene superfamily of intracellular lipid binding proteins that also comprises other groups, including retinol binding proteins, sterol carrier proteins [12]–[14]. It is generally believed that FABPs play a primary function in facilitating the uptake and transport of insoluble long chain fatty acids in aquatic cytosol [12]–[16].

Over 400 vertebrate FABPs and nearly 40 invertebrate FABPs have been identified [12]. Although the amino acid sequence identity between vertebrate and invertebrate FABPs is generally low (25–47%), the tertiary structures of all known FABPs are highly conserved [12], [14], [17]. In vertebrates, FABPs have been classified into 12 isoforms according to the tissue where they were initially discovered, such as heart or muscle-type FABP (H-FABP), liver-type FABP (L-FABP) and adipose-type FABP (A-FABP) [14], [18]. In contrast to those of vertebrate FABPs, the pattern of tissue distribution, the biological role, the tertiary structure, and the ligand binding property of invertebrate FABPs remain mostly unknown. In insects, FABPs have only been reported in muscles, midgut and teratocytes [12], [19]. Typically, FABPs are expressed at high levels in tissues with active lipid metabolism, such as mammalian livers and insect flight muscles [14], [20]. In these tissues, FABPs are thought to play an important role in supplying fatty acids for lipid metabolism [21]–[23].

In this study, we described the discovery and the characterization of a novel FABP from the light organ of adult firefly, *Luciola cerata*. The cDNA of this firefly FABP (lcFABP) was successfully cloned from the light organ, and overexpressed in *Escherichia coli*. The structural and ligand binding features of lcFABP were determined. Moreover, the tissue-specific expression and intracellular localization of lcFABP were also investigated.

## Results

### Identification of lcFABP

In order to search for the light organ-specific proteins, tissue homogenates extracted from the light organ and other body segments, including the head, the thorax and the dorsal organ of *L. cerata*, were prepared for the SDS-PAGE analysis (Fig. 1A). Two abundant proteins with molecular weight (MW) of ∼75 kDa and ∼14.5 kDa were found specifically present in the light organ (left panel in Fig. 1B). Among these two light organ-specific proteins, only the 14.5 kDa protein was successfully identified by Edman degradation. The N-terminal sequence of the 14.5 kDa protein was determined to be ‘VQLAGTYKLEKNENFEEY’. A homology search for this sequence in the NCBI protein database matched the proteins belong to FABP superfamily with a sequence identity up to 78%. Thus, this 14.5 kDa protein is named as *L. cerata* fatty acid-binding protein, lcFABP.

### Cloning and sequence analysis of lcFABP

**Figure 2:**
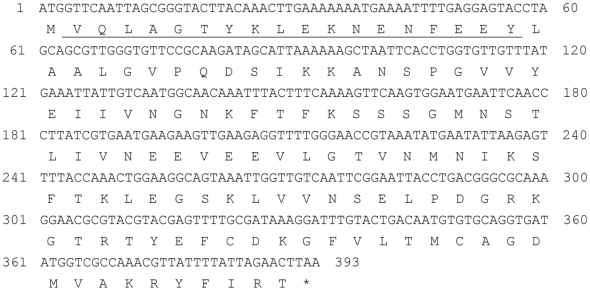
Nucleotide and deduced amino acid sequences of lcFABP. The N-terminal amino acid sequence determined by Edman degradation is underlined. A start codon ATG and its corresponding methionine are assigned to the first position of the sequences.

To obtain the full-length cDNA of lcFABP, reverse transcription polymerase chain reaction (RT-PCR) was performed using a modified oligo (dT)-adaptor primer as the reverse transcription primer, and a degenerated lcFABP forward primer and adaptor reverse primer. A DNA fragment of approximately 500 bp was amplified in RT-PCR (data not shown). For further recombinant protein expression, an ATG codon was assigned to the first position of lcFABP cDNA as initial methionine residue (Fig. 2). The lcFABP protein sequence deduced from its cDNA contains 130 amino acids with a predicted MW of 14.448 kDa (Fig. 2). BLASTP analysis (http://blast.ncbi.nlm.nih.gov/Blast.cgi) of the completed lcFABP protein sequence revealed the highest identity of 47% to a hypothetical fatty acid-binding protein (FABP) from the red flour beetle, *Tribolium castaneum* (accession no: XP969762).

**Figure 3:**
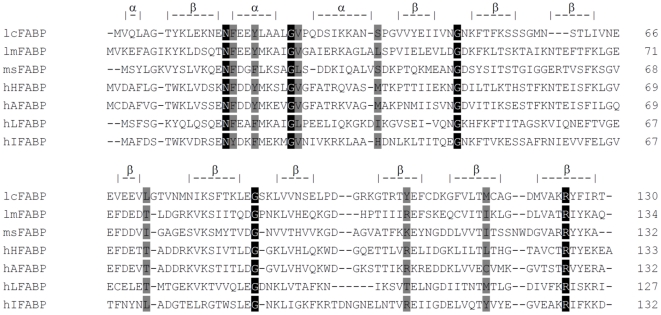
Multiple amino acid sequence alignment of lcFABP and other FABPs from insects and human. The FABP sequences aligned with lcFABP include: lmFABP from locust, *L. migratoria* (GenBank accession no: AAB30739); msFABP from moth, *M. sexta* (AAA29314); hHFABP from human heart (NP004093); hAFABP from human adipocytes (NP001433); hLFABP from human liver (AAA52418); hIFABP from human intestine (P12104). The lcFABP reveals a sequence identity of 31% to lmFABP, 26% to msFABP, 30% to hHFABP, 29% to hAFABP, 30% to hLFABP, 29% to hIFABP. The conserved residues are shaded in black, and the residues involved in oleic acid binding of lmFABP are highlighted in gray. Denoted secondary structures on the top of panel (α: alpha-helix; β: beta-sheet) are derived from lmFABP. The alignment was performed using ClustalW, v2.0.

Amino acid sequence alignment of lcFABP and representative FABPs from insects and human shows that five amino acid residues (an asparagine, three glycines and an arginine) are commonly shared between lcFABP and other FABPs (Fig. 3, shade in black). In addition, the sequence of lcFABP shows an identity range of 26–31% to the aligned FABPs (Fig. 3). Among these aligned FABPs, lcFABP reveals the highest sequence identity of 31% to lmFABP (a locust FABP). Four of the eight lmFABP amino acid residues involved in fatty acid binding (Fig. 3, shade in gray) are also found to be conserved in lcFABP [24].

### Fatty acid binding property of recombinant lcFABP

The binding property of lcFABP to fatty acids was analyzed by a fluorescence displacement assay using 1-anilinonaphthalene-8-sulfonic acid (ANS) as fluorescent probe [25]. This assay utilizes ANS's property of emitting intense fluorescence when bind to the hydrophobic region of protein non-covalently. The displacement of ANS from ligand binding site of FABPs by fatty acid or other hydrophobic ligand will result in the reduction of fluorescence. In this way, the apparent inhibition constants (Ki) of fatty acid or hydrophobic ligand to FABPs can be measured [19], [25]-[27].

**Figure 4:**
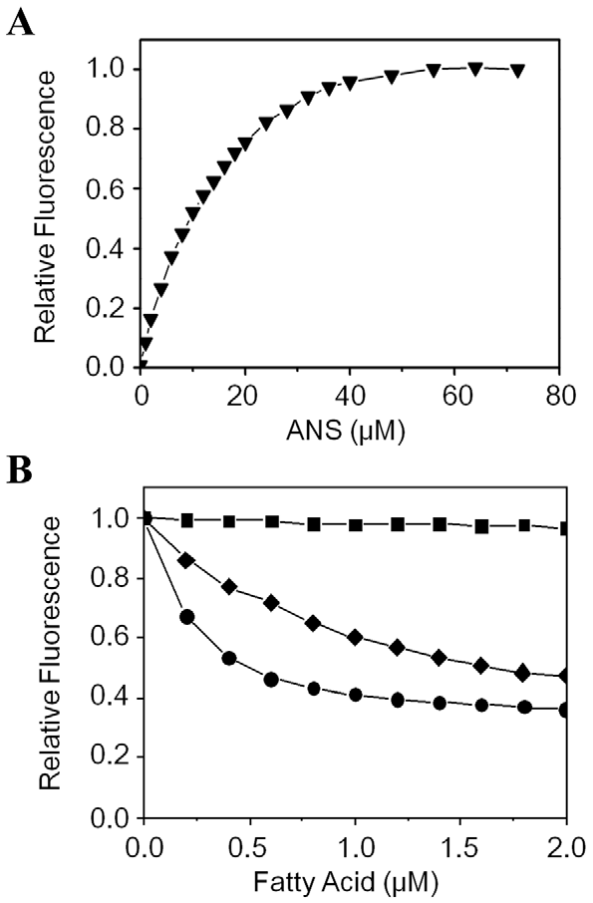
Fluorescence binding and displacement assay for recombinant lcFABP. A) Recombinant lcFABP was titrated with fluorescent probe ANS (▾), and increase in fluorescence was recorded until saturation. B) Displacement of bound ANS from lcFABP by fatty acids. The pre-mixed recombinant lcFABP and ANS was titrated with increasing concentration of fatty acids: C_10:0_, capric acid (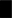); C_16:0_, palmitic acid (•); and C_20:4_, arachidonic acid (⧫), and the reduction of fluorescence was recorded until saturation. The detailed procedures has been described in the Experimental Methods. The results are taken from a single trial and are representative of at least three independent trials.

Recombinant lcFABP was overexpressed in *E. coli* cells, and purified by a two-step chromatography for ligand binding assay. Initially, the binding of ANS to lcFABP was investigated by a fluorescence titration (Fig. 4A), and an apparent dissociation constant (Kd) of 11.16±1.50 µM for ANS-lcFABP complex was calculated by analyzing the binding data. After that, the binding of fatty acid to lcFABP was examined by a competitive displacement titration. The results demonstrated that different fatty acids have distinct capabilities to displace ANS from lcFABP (Fig. 4B). Hence, fatty acids with carbon chain length ranging in C_10_-C_20_ were extensively examined, and their Ki were calculated (Table 1). The results showed that the lcFABP binds strongly (average Ki<1 µM) to steric acid (C_18:0_) with Ki of 0.25±0.01 µM, and to palmitic acid (C_16:0_) with Ki of 0.55±0.12 µM, and to myristic acid (C_14:0_) with Ki of 0.64±0.14 µM, respectively. Conversely, the lcFABP binds moderately to saturated dodecanoic acid (C_12:0_), monounsaturated oleic acid (C_18:1_) and polyunsaturated arachidonic acid (C_20:4_), and poorly to the saturated capric acid (C_10:0_). These binding studies confirmed that the recombinant lcFABP is a functional protein for fatty acids binding as other reported FABPs [12], [16].

**Table 1:** Binding of lcFABP to various fatty acids was determined by fluorescence (ANS) displacement assay.

Fatty acid ligand	Ki (µM)
Capric acid (C_10:0_)	118.15±6.24
Dodecanoic acid (C_12:0_)	1.67±0.15
Myristic acid (C_14:0_)	0.64±0.14
Palmitic acid (C_16:0_)	0.55±0.12
Steric acid (C_18:0_)	0.25±0.01
Oleic acid (C_18:1_)	1.00±0.17
Arachidonic acid (C_20:4_)	1.59±0.16

In this study, we also investigated whether lcFABP could bind firefly luciferin using ANS displacement assay (Fig. S2). Luciferin emitted a strong and stable intrinsic-fluorescence that was independent from the addition of palmitic acid or ANS (data not shown). After subtracting the intrinsic-fluorescence of luciferin, the result demonstrated that the addition of luciferin to ANS-lcFABP complex (Fig. S2, black trace at 200s and 300s) or to palmitic acid-lcFABP complex (Fig. S2, gray trace at 400s) reduced as much as about 12% of fluorescence. Compared with the displacement effect of palmitic acid (Fig. S2, black trace at 400s and gray trace at 200s and 300s), which can reduce as much as 40% of fluorescence, the result indicated that lcFABP interacts weakly with luciferin relative to palmitic acid.

### Tissue-specific distribution and intracellular localization of lcFABP

Western blotting analysis using a rabbit antiserum specifically against the N-terminal sequence of lcFABP revealed an intense lcFABP signal in the light organ, and a weak signal in the dorsal organ, but none in the head and the thorax (right panel in Fig. 1B). This result is consistent with silver-stained SDS-PAGE analysis (left panel in Fig. 1B). Densitometric quantification assay (see Fig. S1) showed that lcFABP constituted 14.97±2.29% of total soluble proteins in the light organ (average from four samples).

**Figure 5:**
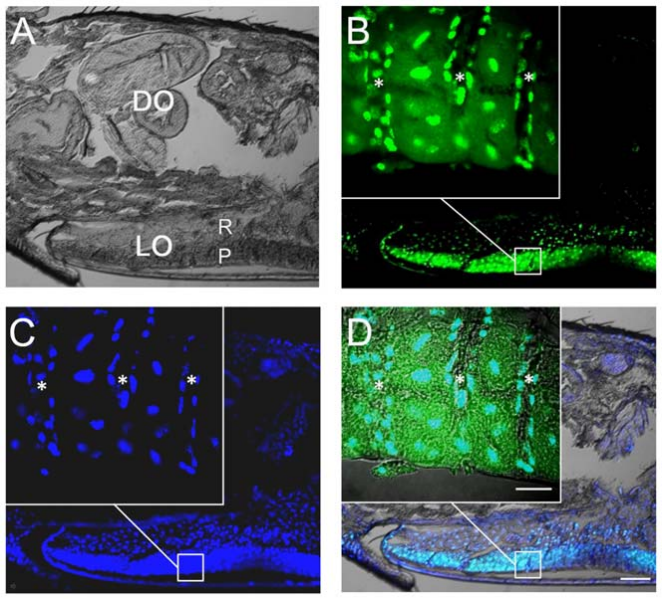
Immunolocalization of lcFABP in the light organ of *L. cerata*. A) DIC micrograph of a transverse tissue section prepared from the 6^th^ body segment of *L. cerata* shows the abdominal light organ (LO) consists of a photogenic layer (P) and a reflector layer (R). Dorsal organ (DO), located at the dorsal site of light organ, contains various tissues. B) Confocal micrograph of the same section using specific antiserum reveals the abundantly expression and distribution of lcFABP in photogenic layer (inner panel) of light organ. C) Confocal micrograph of nucleus staining with DAPI. D) Merging micrograph of A, B and C. Three tracheal trunks were marked with *. Scale bar, 100 µm. Inset scale bar, 20 µm.

Confocal laser microscopy using double staining against lcFABP (green in Fig. 5B) and nucleus (blue in Fig. 5C) revealed the detailed distribution of lcFABP in the light organ. Upon observation in both DIC (Fig. 5A) and DAPI staining of the nucleus (Fig. 5C), the ventral photogenic layer (P) and the dorsal refractory layer (R) can be clearly demarcated. The most intense lcFABP signal was detected in the photogenic layer (Fig. 5B). High magnifying observation of the photogenic layer revealed that lcFABP presents in all cells (inner panel in Fig. 5B). Cells about 20 µm in size localized between the tracheal trunks (marked in * at the inner panels in Fig. 5B, C and D) should be the light-producing cells-photocytes, comparing to the anatomy in other Asiatic fireflies [3]. The co-localization of lcFABP and DAPI in the photocytes (Fig. 5D) indicated the presence of lcFABP in both cytosol and nucleus.

## Discussion

Many fireflies, including *L. cerata,* do not feed but take only water in their adult stage [1]. Therefore, it is possible that the lipid stored in larval stage is the major energy source for adult fireflies during their two to three week life span. The utilization of lipid storage is an intricate process involving a series of protein-mediated processing and delivery of lipid, and has been extensively studied in locusts and moths [22]. However, no study regarding to the lipid utilization or its related-protein was reported in fireflies previously. In this study, the novel firefly protein (lcFABP) that we found in the light organ is believed to be an important carrier involved in intracellular lipid mobilization, and might be functionally linked to photocytes. To our knowledge, this is the first report of the presence of a FABP in bioluminescent tissues.

The lcFABP was originally identified by N-terminal sequencing, and successfully isolated its cDNA from the light organ of *L. cerata*. The lcFABP cDNA encoded a protein of 130 amino acids, similar in size with other reported FABPs (126-134 amino acids) [16]. In North American firefly, *Photinus pyralis,* its light organ contains an abundant protein similar in molecular weight to that (∼14.5 kDa) of lcFABP [6], suggesting that the FABPs might also present in other firefly species.

In the amino acid sequence alignment of lcFABP and other FABPs from insects and human, we found that the most significant structural feature shared between lcFABP and other FABPs is the positively-charged arginine residue located at the C-terminal end of FABPs (Fig. 3, shaded in black). This arginine residue is a key amino acid in the ligand binding pocket of FABPs for the electrostatic interaction with the carboxyl group of the fatty acid [28]. In addition, we also found common shared features between lcFABP and the locust FABP (lmFABP), including the amino acid residues (Fig. 3, shaded in gray) interacting with the methylene group of the hydrocarbon chain of fatty acid [24], and the three unique glycine residues located at the hinge region between alpha-helix and/or beta-sheet (Fig. 3, shaded in black). In human FABP (I-FABP), these glycines are essential for maintaining the stability of protein conformation [29]. Presumably, the tertiary structure of lcFABP might contain the conserved structure with a beta-barrel linking to a helices cap as reported in all other FABPs [12], [14].

We confirmed the fatty acid binding ability of recombinant lcFABP using a fluorescence (ANS) displacement assay (Fig. 4A and B). The results demonstrated that lcFABP binds strongly to the saturated long chain fatty acids (C_14:0_-C_18:0_) much higher than other examined fatty acids, including unsaturated arachidonic acid (C_20:4_) and oleic acid (C_18:1_), saturated capric acid (C_10:0_) and dodecanoic acid (C_12:0_) (Table 1). Similar ligand preference has also been described in the study of aphid FABP [19]. In addition, we also found that the lcFABP binds weakly to luciferin. However, whether this binding is specific still require further investigation.

Through a tissue distribution screening, we found that lcFABP was specifically and abundantly expressed in the light organ (Fig. 1B). This finding implied that the light organ might be the most lipid metabolic-active tissue in *L. cerata*. We estimated that the lcFABP constitutes about 15% of total cytosolic proteins in the light organ. Similar high FABP content (approximately 18% of total cytosolic proteins) has only been reported in flight muscles of adult desert locust, *Schistocerca gregari*a [20]. In other insect and vertebrate tissues, FABPs only make up about 1% to 11% of total cytosolic proteins [14], [30], [31]. The presence of high FABP content in locust flight muscles was thought to maintain efficient energy source supply during the sustained flight in long distance migration [32]. Moreover, it has been shown that the amount of FABP is correlated to the lipid metabolic rate in locust and vertebrate muscles [15]. Firefly light organ is generally believed to be differentiated from fat body [9], but in physiological and anatomical features, the light organ resembles muscle tissue more than fat body. For examples, both light organ and muscle tissue are abundantly supplied with nerve fibers, and both tissues consume ATP for sustained activities (flashes or flight). Considering the similarity of lcFABP and locust flight muscle FABP in structural features (Fig. 3) and abundance within the respective organ, the role of lcFABP in light organ might similar to that of locust FABP in flight muscle.

In the light organ of *L. cerata*, the most abundant lcFABP was detected in cells of the photogenic layer (Fig. 5B). We found that both cytosol and nucleus of photocytes contained abundant lcFABP. Similar intracellular localization has been reported in locust flight muscle, hepatocytes and adipocytes [15]. The size (14∼15 kDa) of FABPs is considered to be small enough to pass through nuclear pores [15]. Many vertebrate FABPs (L-FABP, H-FABP, A-FABP and E-FABP) have been found to be able to interact with transcription factors such as peroxisome proliferator -activated receptors (PPAR) in nucleus, thus, they are thought to be involved in regulating the expression of genes related to lipid metabolism [15], [33]. The accumulation of the lcFABP in nucleus (Fig. 5B, C and D) suggested that the protein might also participate in the regulation of gene expression. However, this hypothesis still remains for further investigation.

Considering both mitochondria and peroxisome are the organelles responsible for lipid metabolism [34], the lcFABP in cytosol of photocytes (Fig. 5B) might be functionally linked to these organelles. Firefly luciferase locates and functions in the peroxisome of photocytes [35]–[37]. The luciferases of *P. pyralis* and *Luciola cruciata* (a Japanese firefly) were found catalyzing not only the bioluminescent reaction but also the long chain fatty acyl-CoA synthetic reaction [11]. The conversion of long chain fatty acids to fatty acyl-CoA is the initial and essential step in fatty acids metabolism [38]. Whereas the amino acid sequences of the reported luciferases among Asiatic fireflies are highly similar (80.8–90%) [39], it is likely that the unexplored luciferase of *L. cerata* also retains a similar catalytic activity to long chain fatty acids. These evidences suggested that the lcFABP might play a key role in supplying long chain fatty acids to peroxisomes for the luciferase-catalyzed long chain fatty acyl-CoA synthetic reaction in photocytes.

Based on *in vitro* studies, firefly bioluminescence was considered to be a high energy efficient reaction (nearly 88% of quantum yield) [40], but a recent report indicated that the efficiency of the reaction, only about 41% of quantum yield, is not that high as previously thought [40]. In light organ and photocytes, the actual energy demand for sustaining bioluminescent flashes is still unclear. Both lcFABP and locust flight muscle FABP are similar in structural features, abundance and anatomical distribution; and considering the similarity in anatomical and physiological features between light organ and locust flight muscle, suggests lcFABP might be an energy source supplier. Hence, the specific abundance of lcFABP within the light organ implied that sustained bioluminescent flashes in the light organ might be a high energy demanding process.

### Conclusion

We demonstrated that the structural characteristics and ligand preferences of lcFABP are highly conserved to those of other reported FABPs. Significantly, the specific abundance of lcFABP within the light organ suggests that sustained bioluminescent flashes in the light organ might be a high energy demanding process. In the photocytes, lcFABP might play a key role in providing long chain fatty acids to peroxisomes for the luciferase-catalyzed long chain acyl-CoA synthetic reaction.

## Materials and Methods

### Chemical

All chemicals were purchased from Sigma-Aldrich (MO, USA) unless indicated otherwise.

### Firefly source

Male adult fireflies, *Luciola cerata*, for this study, were collected from Nanjhuang township of Miaoli county (Taiwan) after sunset from April to May. Collected specimens were immediately sacrificed with CO_2_ asphyxiation and stored at -80°C before used.

### SDS-PAGE analysis

The light organ (including 6^th^ and 7^th^ body segments) and other body parts (head, throrax and dorsal organ) were dissected from a frozen firefly on ice under a dissecting microscope, and were immediately homogenized in 50 µl cold phosphate buffer contained 50 mM phosphate, 2 mM EDTA, 5 mM β-mercaptoethanol (β-ME), 1 mM phenylmethanesulfonylfluoride (PMSF), pH 6.8. After centrifugation in 13000 rpm for 10 min at 4°C, the supernatant was collected. Protein concentration was measurement by Bradford protein assay [41] using BSA as the standard. The extracted proteins were dissolved in the sample buffer contained 100 mM Tris-OH, 2% SDS, 10% glycerol, 0.002% bromophenol blue, 5 mM β-ME, and followed by heating for 10 min at 100°C before loading to gel. Protein separation was carried out by Sodium dodecyl sulfate-polyacrylamide gel electrophoresis (SDS-PAGE) as described previously [42]. The separated proteins in the gel were stained using PlusOne silver staining kit (GE Healthcare, MD, USA), or directly stained with Coomassie Brilliant Blue, or transferred onto a polyvinylidene difluoride (PVDF) membrane (Millipore, MA, USA) for Western blotting analysis.

### Determination of the N-terminal sequence of lcFABP

The SDS-PAGE separated proteins were transferred to a PVDF membrane with a transfer buffer consist of 50 mM NaBO_2_, 20% methanol, pH 9 in a voltage of 60 V for 1 hr. The transferred proteins were stained by Ponceau S solution, and the protein band of interest at 14.5 kDa was excised from the PVDF membrane. After washed with ddH_2_O for three times, the membrane was dried in air and stored at −20°C before used. The N-terminal sequencing was carried out by Edman degradation using a Procise 494 protein sequencer (Applied Biosystem, CA, USA) by Mission Biotech, Corp. (Taipei, Taiwan).

### Production of lcFABP specific antiserum

The partial N-terminal sequence (VQLAGTYKLEKNENF) of lcFABP was synthesized and used to generate antiserum by MDBio, Inc. (Taipei, Taiwan). Before inject to rabbit, the peptide was conjugated to a carrier protein, Keyhole limpet hemocyanin, for enhancing immunogenicity. Five booster injections with 2 weeks interval were carried out after initial injection. The antiserum of rabbit was collected at 2 weeks after the final immunization. The specificity of rabbit anti-lcFABP serum was determined through comparing with the negative control, rabbit pre-serum by western blotting.

### Western blotting analysis

The SDS-PAGE separated proteins were transferred to a PVDF membrane in the transfer buffer contained 25 mM Tris-OH, 192 mM glycine, 20% methanol, and ran in 60 V for 1 hr. The membrane was then blocked by 5% non-fat milk powder dissolved in phosphate buffered saline (PBS) for 1hr at room temperature (RT). The blocked membrane was probed with rabbit anti-lcFABP serum (1∶1000 dilution with 5% non-fat milk in PBS) at 4°C overnight. After washing with PBS three times (15 min each time), the membrane was then incubated with the 1∶1000 dilution of HRP-conjugated goat anti-rabbit IgG secondary antibody (Invitrogen, CA, USA) for 1 hr at RT. After 3 times washing with PBS, proteins were visualized using with Immobilon Western chemiluminescent HRP substrate (Millipore, MA, USA) and HyBlot CL autoradiography film (Denville Scientific, NJ, USA).

### cDNA library construction

The extraction of total RNA from the light organ was performed using a QIAGEN RNeasy kit (QIAGEN, CA, USA). The fresh light organs dissected from ice-anesthetized fireflies were directly homogenized in the RTL lysis buffer containing 1% β-ME on ice. All the RNA extraction procedures were followed the manufacturer's instruction.

### Primers synthesis for reverse transcription polymerase chain reaction (RT-PCR)

The oligo (dT)-adaptor primer (5′-CAGCAGTGCAGACGCAGAGTATTTTTTTTTTTTTTTT-3′) was synthesized and used as the reverse transcription primer. The adaptor primer (5′-CAGCAGTGCAGACGCAGAGTA-3′) was synthesized to be used as the reverse primer for PCR. According to the determined N-terminal sequence (MVQLAGTY) of lcFABP, a degenerate primer (5′-ATGGTNCARTTRGCNGGWACNTA-3′) was designed and synthesized to be the PCR forward primer, where N means any base, R means A or G, W means A or T.

### RT-PCR amplification of lcFABP gene

For cDNA synthesis, 1 µl of mRNA from the *L. cerata* light organ was reverse transcribed using 1 mM deoxynucleoside triphosphates, 5 units PowerScript Reverse Transcriptase (Clontech, CA, USA), 2.5 pM oligo (dT)-adaptor primer in a 20 µl reaction mixture. The reaction was performed at 42°C for 30 min, 100°C for 5 min and 5°C for 5 min. Polymerase chain reaction (PCR) was then carried out in a volume of 50 µl containing 10 pmol of adaptor primer and lcFABP degenerate primer, 1 unit *Taq* polymerase and 1 µl reverse transcribed product using a PX2 Thermo cycler PCR amplifier with 1 cycle at 95°C for 1 min, followed by 35 cycles at 52°C for 3 min and 72°C for 2 min. The PCR product was visualized on a 1% agarose gel and the DNA fragment of ∼500 bp was recovered using QIAquick Gel Extraction Kit (QIAGEN, CA, USA). The cDNA of lcFABP was then cloned into the yT&A plasmid vector (Yeastern Biotech, Taipei, Taiwan), followed by transformation into competent cells of *E*. *coli*, DH5-alpha (Yeastern Biotech, Taipei, Taiwan) for lcFABP gene sequencing. The full-length cDNA sequence of lcFABP has been deposited to the NCBI GenBank (accession no: JN222802).

### Expression and purification of the recombinant lcFABP

The lcFABP gene was amplified using the forward primer, 5′-AAACATATGGTTCAATTGGCGGGAACGTACAAA-3′, and the reverse primer, 5′-AAACTCGAGTTAAGTTCTAATAAAATAACGTTTGGC-3′ (the *Nde*I and *Xho*I restriction sites are underlined and doubly underlined, respectively). For protein expression, the DNA fragment carrying lcFABP was isolated by digestion with *Xho*I and *Nde*I, and ligated into the pET 23a vector, generating the lcFABP-pET23a plasmid. The lcFABP-pET23a plasmid was then transformed into *E*. *coli*, BL21 RB791 strain. The cells were grown in 2 L L-Broth medium at 37°C supplemented with 50 mg/L ampicillin until 1.0 O.D_600nm_. The expression was carried out at 18°C overnight with shaking in the presence of 1 mM isoprooyl-1-thio-β-D-galactopyranoside (IPTG). The cells were collected by centrifugation in 18000 rpm at 4°C. The cell pellets were resuspended in a buffer containing 50 mM sodium phosphate, 2 mM EDTA, 5 mM β-ME, 1 mM PMSF, pH 6.8. and then disrupted by ultrasonication. After centrifugation in 18000 rpm for 30 min at 4°C, the resulting supernatants were applied to a SP Sepharose High Performance cation exchanger column and AKTA-FPLC (GE Healthcare, MD, USA). The column was pre-equilibrated with an equilibrium buffer (E buffer) containing 50 mM sodium phosphate, 2 mM EDTA, 5 mM β-ME, pH 6. The proteins were eluted at 50 mM NaCl on a 0–500 mM NaCl linear gradient in the E buffer at a flow rate of 1 ml/min. The eluted fractions containing recombinant lcFABP were concentrated using 3 kDa pore size Amicon® Ultra-15 (Millipore, CA, USA) and loaded onto the E buffer pre-equilibrated HiPrep 26/60 Sephacryl S-100 HR gel filtration column (GE Healthcare, MD, USA) at a flow rate of 0.5 ml/min. The eluted fractions containing recombinant lcFABP were concentrated. The purified protein revealed a same MW (∼14.5 kDa) to that of native lcFABP derived from the light organ (see Fig. S1). The protein was stored in E buffer with 0.02% NaN_3_ at 4°C and used within 2 weeks.

### Fatty acid binding assay

Fatty acid binding of recombinant lcFABP was assessed by a competitive displacement assay using fluorescence probe ANS as described previously [25], [27]. F7000 fluorescence spectrophotometer (Hitachi, Tokyo, Japan) was used for measuring the ANS fluorescence signal at the emission 470 nm following excitation at 375 nm. All measurements were carried out in an assay buffer contained 50 mM sodium phosphate, pH 7.5, 140 mM KCl, 5 mM β-ME at 25°C under a dim light. ANS and various fatty acids were prepared freshly in absolute ethanol before used. To evaluate ANS-lcFABP interaction, 2 µM lcFABP in a final volume of 2 ml assay buffer was titrated with 1∼2 ul of ANS from a initial concentration of 0 µM to 80 µM. The apparent dissociation constant (Kd) of ANS-lcFABP complex was measured by nonlinear regression analysis of the binding data as described previously [27]. To evaluated the binding of fatty acids to lcFABP, 2 µM lcFABP was pre-equilibrated with 40 µM ANS for 2 min in assay buffer, and then titrated with increasing concentrations of fatty acids until the reduction of fluorescence became saturate. The apparent inhibition constant (Ki) of each fatty acid to ANS-lcFABP complex was calculated from the displacement data as described previously [27]. All binding experiments were repeated at least three times.

### Confocal microscopy

The 6^th^ body segment of *L. cerata* was dissected and fixed with 4% paraformaldehyde in PBS for 2 hr at RT. After a three times rinse with PBS, the fixed tissue was sunk in PBS containing 20% sucrose overnight at 4°C. Before sectioning, the fixed tissue was placed on a pre-chilled tissue holder and subsequently embedded by O.C.T compound (Tissue-Tek, IN, USA) at −20°C until frozen. Leica CM 3050 cryostat (Leica, Nossloch, Germany) was used for sectioning the embedded tissue into 20 µm thickness at −20°C. The sections were directly attached on a Superfrost/plus slide (Fisher Scientific, PA, USA) and stored at −20°C before used. For immunotissue staining, 2% BSA in PBS was used for blocking the sections and for serum/antibody dilution. After 1 hr blocking at RT, the sections were incubated with rabbit anti-lcFABP serum (1∶1000 dilution) for 1 hr at RT, and rinsing three times with PBS (15 min each time). The DyLightTM 488 label goat anti-rabbit IgG secondary antibody (1∶1000 dilution, KPL, Inc., MD, USA) was added to the sections and incubated for 1 hr at RT. After another three times rinses with PBS, the sections were overlaid with coverslip using Prolong antifade kit (Invitrogen, CA, USA), supplement with 4′,6-diamidino-2-phenylindole (DAPI; Invitrogen, CA, USA) in a 1∶200 dilution. Pre-immune serum or the secondary antibody alone was used as a negative control. The images obtained using Fluoview FV1000 Confocal microscopy (Olympus, Tokyo, Japan), and analyzed using the FV10-ASW V0.1 software (Olympus, Tokyo, Japan).

## Supporting Information

Figure S1 **SDS-PAGE analysis of recombinant lcFABP from** ***E. coli*** **expression and quantification of native lcFABP in the light organ.** The soluble extract of the light organ with a total protein amount of 30 µg (lane: LO), and the purified recombinant lcFABP (lane: 1), and the *E. coli* lysate with IPTG induction (lane: 2), and the *E. coli* lysate without IPTG induction (lane: 3), and the *E. coli* lysate without transformation (lane: 4) were analyzed by SDS-PAGE with a 15% polyacrylamide gel using coomassie blue staining. A densitometric profile (in left panel) corresponding to the light organ (LO) was generated by the Image J program, and used for the lcFABP content quantification. Arrowhead toward right side indicates the densitometric peak or protein band of lcFABP.
(TIF)

Figure S2 **Binding of luciferin to recombinant lcFABP.** Displacement of bound ANS from lcFABP by D-luciferin. Fluorescence change was recorded after successively adding lcFABP (until final concentration become 2 µM at 100 s), D-luciferin (Luc; 1 µM at 200 s and 2 µM at 300 s) or palmitic acid (PA; 1 µM at 200 s and 2 µM at 300 s), and the competitive palmitic acid (2 µM at 400 s) or D-luciferin (2 µM at 400 s) to into the ANS (40 µM) containing buffer using time-dependent model. The results have been subtracted of D-luciferin's intrinsic-fluorescence. All experiments are monitored under a detected wavelength of Ex: 370 nm and Em: 450 nm to reduce the interference of D-luciferin intrinsic-fluorescence to the assay.
(TIF)
