# Supplementary figures and images for: A Photocytes-Associated Fatty Acid-Binding Protein from the Light Organ of Adult Taiwanese Firefly, Luciola cerata
Source: PLoS One. 2011 Dec 29;6(12):e29576. doi: 10.1371/journal.pone.0029576 (PMC3248459; doi:10.1371/journal.pone.0029576)

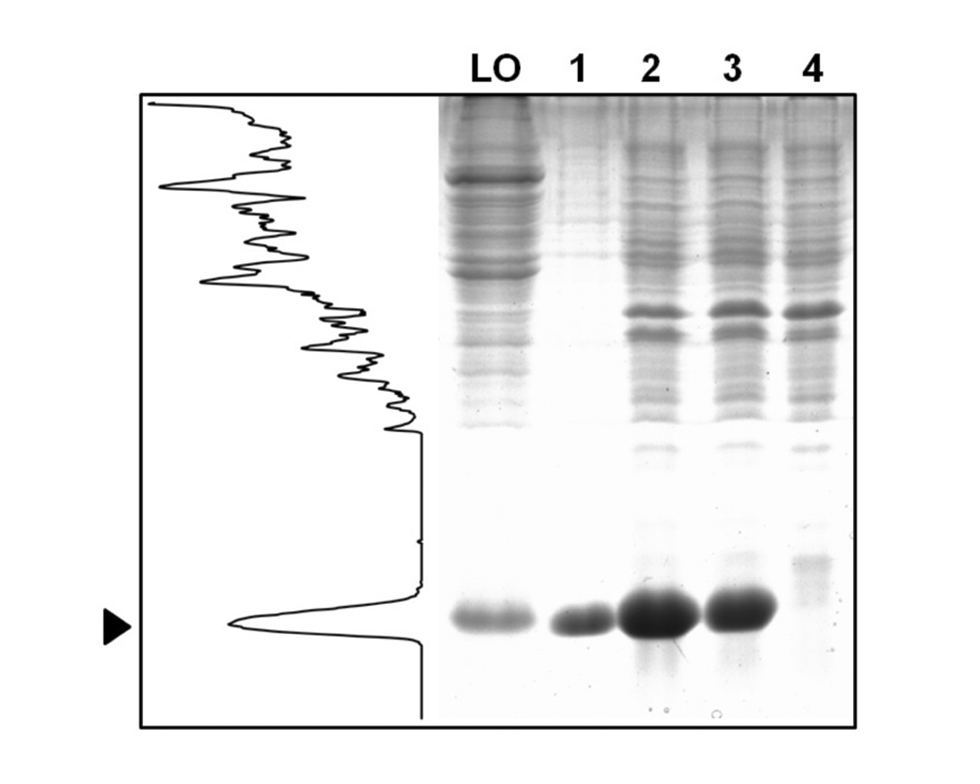

Supplement: Figure S1 — SDS-PAGE analysis of recombinant lcFABP from E. coli expression and quantification of native lcFABP in the light organ. The soluble extract of the light organ with a total protein amount of 30 µg (lane: LO), and the purified recombinant lcFABP (lane: 1), and the E. coli lysate with IPTG induction (lane: 2), and the E. coli lysate without IPTG induction (lane: 3), and the E. coli lysate without transformation (lane: 4) were analyzed by SDS-PAGE with a 15% polyacrylamide gel using coomassie blue staining. A densitometric profile (in left panel) corresponding to the light organ (LO) was generated by the Image J program, and used for the lcFABP content quantification. Arrowhead toward right side indicates the densitometric peak or protein band of lcFABP. (TIF) [file pone.0029576.s001.tif]

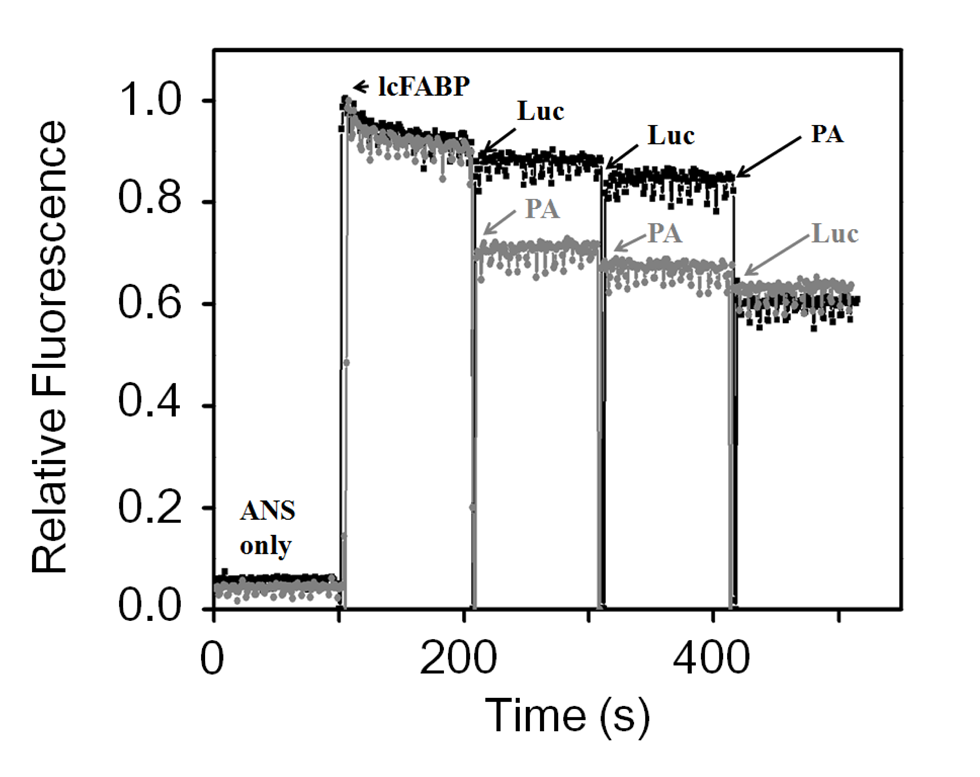

Supplement: Figure S2 — Binding of luciferin to recombinant lcFABP. Displacement of bound ANS from lcFABP by D-luciferin. Fluorescence change was recorded after successively adding lcFABP (until final concentration become 2 µM at 100 s), D-luciferin (Luc; 1 µM at 200 s and 2 µM at 300 s) or palmitic acid (PA; 1 µM at 200 s and 2 µM at 300 s), and the competitive palmitic acid (2 µM at 400 s) or D-luciferin (2 µM at 400 s) to into the ANS (40 µM) containing buffer using time-dependent model. The results have been subtracted of D-luciferin's intrinsic-fluorescence. All experiments are monitored under a detected wavelength of Ex: 370 nm and Em: 450 nm to reduce the interference of D-luciferin intrinsic-fluorescence to the assay. (TIF) [file pone.0029576.s002.tif]
